# Metabolomics in the Diagnosis and Prognosis of COVID-19

**DOI:** 10.3389/fgene.2021.721556

**Published:** 2021-07-23

**Authors:** Mohammad Rubayet Hasan, Mohammed Suleiman, Andrés Pérez-López

**Affiliations:** ^1^Department of Pathology, Sidra Medicine, Doha, Qatar; ^2^Weill Cornell Medical College in Qatar, Doha, Qatar

**Keywords:** COVID-19, SARS-CoV-2, metabolomics, diagnosis, prognosis, volatile organic compounds, mass-spectrometry, nuclear magnetic resonance

## Abstract

Coronavirus disease 2019 (COVID-19) pandemic triggered an unprecedented global effort in developing rapid and inexpensive diagnostic and prognostic tools. Since the genome of SARS-CoV-2 was uncovered, detection of viral RNA by RT-qPCR has played the most significant role in preventing the spread of the virus through early detection and tracing of suspected COVID-19 cases and through screening of at-risk population. However, a large number of alternative test methods based on SARS-CoV-2 RNA or proteins or host factors associated with SARS-CoV-2 infection have been developed and evaluated. The application of metabolomics in infectious disease diagnostics is an evolving area of science that was boosted by the urgency of COVID-19 pandemic. Metabolomics approaches that rely on the analysis of volatile organic compounds exhaled by COVID-19 patients hold promise for applications in a large-scale screening of population in point-of-care (POC) setting. On the other hand, successful application of mass-spectrometry to detect specific spectral signatures associated with COVID-19 in nasopharyngeal swab specimens may significantly save the cost and turnaround time of COVID-19 testing in the diagnostic microbiology and virology laboratories. Active research is also ongoing on the discovery of potential metabolomics-based prognostic markers for the disease that can be applied to serum or plasma specimens. Several metabolic pathways related to amino acid, lipid and energy metabolism were found to be affected by severe disease with COVID-19. In particular, tryptophan metabolism via the kynurenine pathway were persistently dysregulated in several independent studies, suggesting the roles of several metabolites of this pathway such as tryptophan, kynurenine and 3-hydroxykynurenine as potential prognostic markers of the disease. However, standardization of the test methods and large-scale clinical validation are necessary before these tests can be applied in a clinical setting. With rapidly expanding data on the metabolic profiles of COVID-19 patients with varying degrees of severity, it is likely that metabolomics will play an important role in near future in predicting the outcome of the disease with a greater degree of certainty.

## Introduction

The ongoing pandemic of coronavirus disease 2019 (COVID-19) has created massive disruptions and loss of human lives around the world. As of May 2021, the number of laboratory-confirmed cases exceeded 170 million and 3.6 million people succumbed to the disease (WHO, 2020a). The outbreak of COVID-19 emerged at the end of 2019 in Wuhan, China in the form of a series of cases of pneumonia of unknown etiology. The virus causing the disease was soon identified as a novel serotype of coronavirus and was later named as severe acute respiratory syndrome virus 2 (SARS-CoV-2) (Zhou et al., 2020). SARS-CoV-2 is the seventh known coronavirus serotypes that are known to infect humans and the third among the coronaviruses that are known to cause large-scale epidemics. Because of high transmissivity of SARS-CoV-2 and lack of pre-existing immunity to the virus in the population, the virus rapidly spread from its origin to other countries and a global pandemic was declared by the World Health Organization (WHO) in March 2020 (Petersen et al., 2020; WHO, 2020a). COVID-19 is a disease of the respiratory system, but it may progress to severe, life threatening systemic diseases (UpToDate, 2020). Most patients with COVID-19 are asymptomatic or mildly symptomatic. However, approximately 14% of patients may develop severe symptoms and up to 5% of cases could be critical with an overall case fatality rate of 2.3%. In the absence of specific clinical and radiographic features, COVID-19 cannot be reliably distinguished from other respiratory tract infections (UpToDate, 2020, 2021). Therefore COVID-19 can only be confirmed by laboratory diagnosis.

Since the beginning of the pandemic, laboratory testing of suspected individuals for COVID-19 and isolation of positive cases have been the main public health strategies to prevent the spread of the disease. Laboratory diagnosis of viral respiratory tract infections is, in principle, based on viral culture, electron microscopy (EM), antigen detection, serology and nucleic acid amplification tests (NAAT). However, viral culture and EM are now rarely used in clinical diagnostic settings because of longer turn-around time (TAT), and the requirement of intense labor and technical expertise to perform the tests. The clinical usefulness of serology is also very limited because in most cases pathogen specific antibodies may take weeks to be detectable and may require both acute and convalescent sera to make a diagnosis. Antigen detection by direct fluorescent antibody tests (DFA) or rapid immunoassays are available for some common respiratory viruses such as influenza and respiratory syncytial virus (RSV) with variable sensitivity and specificity. However, in recent years, molecular test methods have evolved substantially and have become the test of choice for the detection of a wide range of respiratory viruses including coronaviruses (Thomson, 1999; Das et al., 2018).

Detection of viral RNA in upper respiratory tract specimens, most importantly, in nasopharyngeal swab specimens by NAAT is the standard diagnostic test method for COVID-19. Soon after the genome sequence of SARS-CoV-2 was available, reverse transcriptase quantitative polymerase chain reaction (RT-qPCR) based assays to detect SARS-CoV-2 RNA were developed and were made publicly available on an emergency basis (WHO, 2020b). This was soon followed by rapid development of numerous laboratory-developed and commercial assays for molecular detection of SARS-CoV-2, many of which obtained approval from the Food and Drug Administration (FDA) under emergency use authorization (Ravi et al., 2020). Yet a massive surge in COVID-19 testing and increased demands led to a shortage of test reagents and kits worldwide (ASM, 2020). Therefore, many modifications of the standard test methods have been proposed and evaluated in order to save scarce resources such as direct RT-qPCR of NP swab collected in viral transport medium or in saliva specimens skipping the RNA extraction step or pooled specimen testing of asymptomatic individuals etc. (Bruce et al., 2020; Deka and Kalita, 2020; Hasan et al., 2020; Kriegova et al., 2020; Graham et al., 2021; Vogels et al., 2021). Molecular test methods with alternative chemistry and detection methods such as isothermal amplification or CRISPR-based test methods were developed, and their performance characteristics were compared to the standard test methods (Broughton et al., 2020; Yan et al., 2020; Fozouni et al., 2021; Nouri et al., 2021). These alternative test approaches were designed to be inexpensive, rapid and suitable for point-of-care (POC) applications.

Compared to molecular test methods, the role of SARS-CoV-2 antigen-based methods in managing COVID-19 pandemic was very limited. Although, in principle, antigen tests could have replaced viral RNA detection methods and could have offered faster and easier detection of the virus in POC setting, these tests were not recommended because of poor sensitivity (Scohy et al., 2020). There was also a substantial delay in the commercial development and market release of approved antigen tests for COVID-19. However, a year into the pandemic, with several COVID-19 antigen tests now approved with superior sensitivity than those described earlier, the role of rapid antigen tests is now better recognized, in particular, because of its potential for use in mass screening of symptomatic individuals. To make the best use of antigen tests, a combinatory approach including molecular or antibody tests has also been suggested (Peeling and Olliaro, 2021; Peeling et al., 2021). In parallel with antigen tests, serologic tests to detect antibodies against SAR-CoV-2 in blood have also been developed and many such tests are now commercially available. Because of the delay in the production of detectable antibodies in COVID-19 patients, antibody tests have limited role in acute care setting. However, antibody tests are helpful in detecting past infection or confirming diagnostically challenging COVID-19 cases. Antibody tests are also important for seroprevalence studies, test convalescence sera and to monitor vaccine response.

Besides the paramount roles of molecular and immunological assays, other laboratory parameters have demonstrated to have a reliable adjunctive value in assisting clinicians to predict the course and prognosis of COVID-19, given its broad clinical manifestations ranging from asymptomatic infection to rapid progression to acute respiratory distress syndrome and fatal outcome. For example, some sustained abnormalities in white blood cells, specifically lymphopenia, consistently correlates with a worse outcome in hospitalized adults. Both adults and children with severe COVID-19 show consistent trends toward elevated lactate dehydrogenase (LDH), C-reactive protein (CRP), procalcitonin (PCT), and D-dimer levels (Ponti et al., 2020; UpToDate, 2020). In fact, the growing interest in the use of serial measurements of PCT to predict COVID-19 course in hospitalized patients deserves particular mention. Although levels of PCT are typically normal or minimally elevated in COVID-19 patients on admission, the progressive increase of serum PCT levels has been accurately associated with worse disease prognosis and secondary bacterial infection (Hu et al., 2020; Liu et al., 2020). Of note, the elevation of creatine kinase-myocardial band (CK-MB) and troponin has been consistently reported elevated in adults and children with acute cardiac injury as a result of SARS-CoV-2 infection (Shi et al., 2020). In addition, the progressive derangement of other biochemical parameters such as urea nitrogen, creatinine, liver enzymes, and ferritin, and coagulation parameters such as prothrombin time (PT) and activated partial thromboplastin time (aPTT) has also been linked to the development of severe forms of COVID-19, including multisystem inflammatory syndrome in children (MIS-C), and worse prognosis (Bonetti et al., 2020; Dufort et al., 2020).

In addition to the applications of test methods and approaches that are commonly applied to the diagnosis and prognosis of infectious diseases, COVID-19 pandemic also triggered urgent research on alternative ways including metabolomics-based approaches for the diagnosis and prognosis of COVID-19. The term ‘metabolomics’ was first termed in 1998. Metabolomics is a relatively new field in systems biology after genomics, transcriptomics and proteomics (Kell and Oliver, 2016; Alseekh and Fernie, 2018). Interests in metabolomics have grown in the last 20 years in order to understand the biological processes at the functional level and to a greater depth than those offered by other ‘omics’ approaches. Metabolomics approach is applied to identify metabolites in biological samples both under normal conditions as well as altered physiological states such as under diseased conditions and to determine their association with the altered physiological or pathophysiological process (Klassen et al., 2017). The definition of the term ‘metabolome’ or ‘metabolite’ is broad and may include small to large biomolecules and from primary, endogenous metabolites to secondary, exogenous metabolites. The endogenous metabolome is considered to be highly conserved at the organism level but can be varied by many internal or external variables (Wishart, 2019). Dysregulated metabolic pathways are linked to many human diseases. Therefore, metabolomics-based, metabolite profiling have been increasingly applied in the past two decades to understand the pathophysiology of the disease and to identify biomarkers for the diagnosis and prognosis of the disease (Jacob et al., 2019; Wishart, 2019). The list of human diseases and the list of specimen types for which metabolomics may be applied as a laboratory diagnostic tool is increasing at a rapid pace with the discovery and validation of newer biomarkers. The potential application of metabolomics in the diagnosis and prognosis now goes beyond chronic metabolic diseases to a wide spectrum of diseases including infectious diseases (Wishart, 2019; Evans et al., 2020). Over the past year, a lot of energy was put in research on the application of metabolomic approaches for the diagnosis and prognosis of COVID-19, in an effort to mitigate the crisis arising from the uncertainties associated with the clinical course of the disease and with the inadequacy of sufficient diagnostic and prognostic tools. In this review, we summarize the findings of these studies and discuss the potential and the appropriate context where metabolomics-based approaches may be successfully applied in the diagnosis and management of COVID-19.

## Clinical Metabolomics Analytical Tools

Metabolomics approach, in general, applies the tools of analytical chemistry such as nuclear magnetic resonance (NMR) and mass spectrometry (MS) coupled to different chromatographic methods to measure and characterize the metabolome. Both targeted and untargeted approaches are used depending on the applications (Roberts et al., 2012; Schrimpe-Rutledge et al., 2016). In targeted metabolomics, pre-defined and annotated chemical substances are measured based on standardized procedures. Analysis can be quantitative or semi-quantitative and may include from few to hundreds of metabolites. On the other hand, with untargeted approach, a comprehensive analysis is done to measure all detectable metabolites in a given specimen including unknown metabolites. The large amount of raw data generated from untargeted analysis must be coupled to extensive data processing and statistical validation to generate clinically significant results.

In principle, body fluids such as serum, plasma, urine, cerebrospinal fluid (CSF) and pleural fluid, swab or aspirate from a body site or a tissue specimen are processed to quench and extract metabolites and separate them by applying a variety of chromatography techniques. Most often these separation techniques are combined with the analytical tools or platforms such as MS coupled with liquid chromatography, gas chromatography, ion chromatography or capillary electrophoresis (CE). With recently developed ambient ionization mass spectrometry, clinical specimens can now be directly analyzed with little or no pre-treatment, widening the clinical applications of MS, including POC applications. Currently, MS-based clinical applications mostly rely on electrospray ionization (ESI) and matrix-assisted laser desorption/ionization (MALDI) mass spectrometry for larger biomolecules, and atmospheric pressure chemical ionization (APCI) and ambient ionization (AI) for smaller metabolites. For simultaneous analysis of multiple analytes, liquid chromatography-tandem mass spectrometry (LC-MS/MS) has emerged as a powerful technique. Rapid identification of microbes, newborn screening (NBS), therapeutic drug monitoring and measurement of vitamin D levels are some examples of MS based applications that are approved by US FDA and are in clinical use (Banerjee, 2020).

Apart from MS techniques, the other analytical tool used in metabolomic studies is NMR spectroscopy. NMR spectroscopy generates structural information of biomolecules based on differences in the energy levels of ^1^H, ^13^C, and ^31^P nuclei (Collino et al., 2013). Databases of reference NMR spectra for hundreds of metabolites are evolving and are being made freely accessible. Proton (^1^H) NMR spectroscopy is the most common method applied in metabolomics studies. NMR spectroscopy can be applied to profile hundreds of metabolites in an unbiased and comprehensive manner. Unlike MS based methods, NMR spectroscopy requires little or no sample pre-treatment and is therefore non-destructive in nature. As a result, in addition to biofluids or tissue extracts, NMR can also be applied to solid tissues and organs for real time data. NMR spectroscopy does not require chromatographic separation of metabolites for analysis. NMR can generate quantitative data and has high reproducibility, but the sensitivity is much lower than the MS methods (Emwas et al., 2019). At present, the clinical laboratory application of NMR is limited to profiling of lipoproteins for the assessment of patients at risk for atherosclerotic cardiovascular diseases and analysis of fecal lipids for the diagnosis of pancreatic or intestinal disorders (Mayo Clinic Laboratories, 2021).

## Metabolomics in Infectious Disease Diagnosis and Prognosis

Metabolomics is a very early stage, emerging area of science for infectious disease diagnostics. Conventionally, the diagnosis of infectious diseases relies on direct methods such as the detection of the pathogens by microscopy, culture, antigen tests or NAAT or by indirect methods such as antibody tests. Apart from pathogen detection, infectious disease diagnosis also frequently depends on other laboratory parameters such as blood cell count, erythrocyte sedimentation rate (ESR), presence of neutrophils in the potentially infected body sites and non-specific inflammatory biomarkers such as CRP and PCT. In principle, metabolomics-based approaches can be applied to detect pathogens directly or by targeting host-response biomarkers that are specific to certain infections. Microbial identification by matrix assisted laser desorption ionization-time of flight mass spectrometry (MALDI-TOF MS) is an example by which pathogens are directly identified based on their characteristic proteomic fingerprints. MALDI-TOF MS is an FDA approved technology for pathogen identification that has been widely used since it is introduction in the clinical Microbiology laboratories worldwide. It has been proven as a rapid, sensitive and cost-effective method in the identification of bacterial, fungal, and mycobacterial pathogens from cultures and directly from clinical samples such as positive blood cultures and urine samples (Bille et al., 2012; Wieser et al., 2012). Also, MALDI-TOF has been used for susceptibility testing and to detect resistant markers in some bacterial and fungal isolates (Sauget et al., 2018; Peng et al., 2019).

Apart from MALDI-TOF MS applications, other MS techniques such as LC-MS/MS, GC-MS and tandem MS are limited to research setting. While LC-MS/MS is the most commonly used method for other clinical applications, it is not yet routinely used in clinical microbiology laboratories. However, potential application of LC-MS/MS in identifying bacteria and antibiotic resistance are increasingly being studied (Fleurbaaij et al., 2015; Nomura et al., 2020). Studies on the application of NMR spectroscopy in clinical microbiology, in particular, direct detection of pathogens is also very limited. Recently NMR spectroscopy has been applied in a study to identify and quantify urinary pathogens based on specific metabolite profiles for the diagnosis of urinary tract infections (Capati et al., 2017). Both MS and NMR spectroscopy were applied to identify specific biomarkers that can discriminate patients with *Clostridium difficile* infections, enteric fever caused by *Salmonella* species, melioidosis caused by *Burkholderia pseudomallei* and tuberculosis caused by *Mycobacterium tuberculosis* from healthy individuals. Metabolomics studies on parasitic infections such as malaria caused by *Plasmodium* species and a few studies on invasive fungal infections have also been reported. GC-MS was applied for diagnostic staging of malaria infection in children showing the increased presence of long-chain fatty acids and other organic acids in patients with severe malaria, and a reduction of amino acids and increased sugar levels in mild cases. In another study, plasma samples from patients with onchocerciasis or ‘river blindness’ were analyzed by non-targeted capillary electrophoresis time-of-flight mass spectrometry (CE-TOFMS) identifying metabolites such as serotonin, hypoxanthine, pipecolic acid and inosine as potential biomarkers of the disease (Bennuru et al., 2017). For viral infections, metabolomics-based biomarker studies were mainly focused on viruses that causes systemic infections such as human immunodeficiency virus infection (HIV), hepatitis C infection, and dengue fever (Fernández-García et al., 2018). In a few studies, MS based proteomic approaches were applied to detect respiratory viruses such as influenza virus and human metapneumovirus either from cultured cells or directly from nasopharyngeal swab (NPS) specimens (Foster et al., 2015; Majchrzykiewicz-Koehorst et al., 2015).

Recently, research on metabolomics based diagnostic and prognostic biomarkers for infectious diseases has been accelerated by the urgency and the magnitude of the ongoing COVID-19 pandemic. These studies include efforts to develop novel, inexpensive and simple diagnostic applications as well as efforts to enhance knowledge on the biomarkers to predict the outcome of the disease.

## Metabolomics in COVID-19 Diagnosis

### Analysis of Volatile Organic Compounds (VOCs) in the Exhaled Air

Human exhaled air contains a great number of volatile organic compounds (VOC), which are organic compounds that have high vapor pressure at room temperature and are therefore emitted into the air. VOCs in human exhaled air can be quantified using gas chromatography – mass spectrometry (GC-MS) and through the use of sensors known as electronic nose (eNose) (Kuo et al., 2020). There are also reports of the use of trained, sniffing dogs to detect VOCs associated with human diseases. Because of a greater understanding of the composition of VOCs in the exhaled breath of healthy subjects and those under diseased states, human breathomics has emerged as a branch of metabolomics. A comprehensive human breathomics reference database containing data on 913 VOCs from 2766 published literature was developed, which are linked to several respiratory diseases such as asthma, chronic obstructive pulmonary disease (COPD) and cystic fibrosis (Kuo et al., 2020). Exhaled air is considered as a very convenient specimen for respiratory diseases including viral infections because of the non-invasive nature and the ease of collection of the specimen (Davis et al., 2021). However, studies with breath VOCs associated with respiratory viral infections were limited to human rhinovirus and influenza virus in cultured cells (Aksenov et al., 2014; Schivo et al., 2014). During the past year, because of massive surge in demands for COVID-19 testing a renewed interest was noted in breath testing for respiratory viral infections. Detection of COVID-19 associated VOCs in breath was considered as one of the most appealing approach for mass-scale screening of individuals in POC setting (Giovannini et al., 2021).

Several pilot studies were conducted to detect and identify COVID-19 associated VOC signatures and to evaluate the potential of the approach for COVID-19 testing in comparison with standard RT-qPCR (Table 1). Most studies used exhaled air as the specimen but other convenient specimen types such as armpit sweat, saliva or used face masks were also tested. Detection methods in these studies varied from different MS techniques to the use of electronic nose or trained, sniffing dogs. The sensitivity of SARS-CoV-2 detection varied from 75 to 100% and the specificity ranged from 66 to 96%. VOCs associated with COVID-19 infection were identified in studies that applied MS methods for analysis. In an interesting proof of concept study, metabolomic analytic approaches were applied to discriminate between COVID-19 acute respiratory distress syndrome (ARDS) and non-COVID-19 ARDS cases based on VOCs in their exhaled air (Grassin-Delyle et al., 2021). By using real-time, online, proton transfer reaction time-of-flight mass spectrometry a VOC breathprint was established to identify COVID-19 ARDS patients requiring invasive mechanical ventilation with high sensitivity and specificity. An untargeted metabolomics strategy with different machine learning algorithms were applied to discover VOCs associated with COVID-19 specific ARDS, and four prominent VOCs methylpent-2-enal, 2,4-octadiene, 1-chloroheptane, and nonanal were found to be present at a significantly high concentration in the breath of COVID-19 ARDS patients. Some of these VOCs are distinct from VOCs detected in non-severe COVID-19 cases in other studies (Berna et al., 2020; Ruszkiewicz et al., 2020) suggesting the potential use of these VOCs as the severity markers of the disease. However, the number of specimens were inadequate in majority of these studies, and further validation of data and standardization of the methods is necessary. Given that a number of clinical trials have so far been registered with US National Library of Medicine for COVID-19 breath analysis and many of them are now actively recruiting participants, it is likely that more data in this regard will be available in near future.

**TABLE 1:** COVID-19 testing by analysis of volatile organic compounds (VOC).

Specimen	No of patients	Reference method	Detection method	Potential breath biomarker	Results	References
Breath sample	98	RT-qPCR	GC-IMS	Ethanal, octanal, acetone, butanone, methanol, heptanal	Sensitivity: 82.4–90% Specificity: 75–80%	Ruszkiewicz et al., 2020
Saliva, tracheobronchial secretions	1012	RT-qPCR	Sniffing dog	–	Sensitivity: 82.6% Specificity: 96.3%	Jendrny et al., 2020
Breath sample	40^1^	RT-qPCR	PTR-TOF MS	Methylpent-2-enal, 2,4-octadiene 1-chloroheptane, and nonanal	Sensitivity: 90% Specificity: 94%	Grassin-Delyle et al., 2021
Armpit sweat samples	177	RT-qPCR	Sniffing dog	–	Success rate: 76–100%	Grandjean et al., 2020
Breath sample	26^2^	RT-qPCR	GCxGC ToF-MS	Octanal, nonanal, heptanal	Sensitivity: 100% Specificity: 66%	Berna et al., 2020
Breath sample	219	RT-qPCR Antibody test	Electronic nose	–	Sensitivity: 86% NPV: 92%	Wintjens et al., 2020
Pharyngeal secretion, face masks, cloths	80	RT-qPCR	Sniffing dog	–	Sensitivity: 86% Specificity: 92%	Eskandari et al., 2021

*RT-qPCR, reverse transcriptase quantitative polymerase chain reaction; GC-IMS, gas chromatography-ion mobility spectrometry; PTR-TOF MS, proton-transfer-reaction time-of-flight mass spectrometer; GCxGC ToF-MS, two-dimensional gas chromatography and time-of-flight mass spectrometry; NPV, negative predictive value. ^1^ARDS patients; ^2^Pediatric.*

COVID-19 detection by sniffing dogs is well suited for field applications, in particular, for large-scale screening of individuals at the airports. The idea of testing human diseases with sniffer dogs, also known as detection dogs, is not new. Dogs have an unusual sense of smell that is 10,000–100,000 times greater than humans (Ellen, 2020). Behind the human nasal cavity are special nerve cells called olfactory receptors. Any chemical that produces odor enters the nasal cavity and sends signals to the brain through these sensor cells to create odor. Dogs are no exception but compared to only 6 million olfactory receptors in humans, dogs have 300 million olfactory receptors. Furthermore, the part of the dog’s brain that possess the sense of smell is 40 times larger than humans. Due to the dog’s extraordinary sense of smell, it is most commonly used at airports, especially in the detection of explosives or illegal drugs (Cambau and Poljak, 2020; Else, 2020). Dogs have also been employed in the detection of VOCs associated with human diseases other than COVID-19, such as cancer, diabetic ketoacidosis, hypoglycemia and infections such as CDI, viral diarrhea, and UTI (Sakr et al., 2021). For COVID-19, although the amount of data published so far is limited, additional trials are currently underway in many countries including United Kingdom (UK) and Chile. A clinical trial registered by Arthropod Control Product Test Centre (ARCTEC), UK is currently recruiting with an estimated, 16250 participants^1^. In United Arab Emirates (UAE), several trials were conducted with K9 police sniffer dogs trained to detect armpits of infected persons with an accuracy rate of 92%, and the country is believed to be the first in the world to implement Dog sniffing method for COVID-19 detection at their airports (MOI, 2020).

### Mass-Spectrometric Analysis of Clinical Specimens

Apart from the analysis of VOCs in the expelled air by COVID-19 patients, studies on COVID-19 detection based on specific metabolic signatures were very limited. In a study on COVID-19 positive or negative intensive care unit (ICU) patients, plasma concentrations of 162 metabolites were assessed by LC-MS/MS and proton NMR. Kynurenine and arginine were among the top performing metabolites to distinguish COVID-19 positive patients from healthy controls as well as COVID-19 negative ICU patients, and arginine/kynurenine ratio accurately predicted COVID-19 disease status irrespective of age, sex, and hospital admission status (Fraser et al., 2020). However, the role of these findings in COVID-19 diagnosis is limited given that only few patients were assessed and that only ICU patients were assessed. In another study, plasma metabolome of a limited number of COVID-19 patients and healthy controls were assessed revealing the role of the cytosine and tryptophan-nicotinamide pathways in discriminating COVID-19 patients from the controls with an accuracy of >74% and the sensitivity and specificity of >75% (Blasco et al., 2020). Similarly, preliminary data from another study show that tryptophan levels lower than 105 μM and kynurenine levels higher than 5.3 μM have an AUC of 0.95 on receiver operating characteristic (ROC) curves to distinguish COVID-19 positive and negative patients (Thomas et al., 2020).

Although metabolite data in relation to COVID-19 diagnosis were limited, a number of studies have been published during the past year on the MS based detection of SARS-CoV-2 offering potential alternatives to molecular tests. Prior to the emergence of COVID-19, MS has rarely been applied to analyze upper respiratory tract specimens such as NPS specimens for proteomic signatures associated with specific viral infections (Foster et al., 2015). In the wake of huge demands for alternative test strategies during the current pandemic, a great deal of effort was put into the use of MS for COVID-19 diagnosis. One of the earliest among these efforts was an attempt to identify and characterize viral proteins from shotgun proteomics dataset acquired on SARS-CoV-2 infected Vero cells that could potentially be used for targeted MS (Gouveia et al., 2020). This study was followed by several proof of principle, targeted proteomic studies aimed at identifying SARS-CoV-2 in nasopharyngeal or oropharyngeal swabs and even in gurgle solutions (Table 2). In most studies peptides originating from the viral nucleoprotein (N) were identified as potential targets for LC-MS or LC-MS/MS based testing because of the abundance of the protein. Most of these studies were preliminary either because no clinical validation was done, or because only a limited number of clinical samples were used for validation. Furthermore, complex pre-processing steps including precipitation, lysis, digestion and purification of peptides limits the practicality of these methods for routine use. However, in one of the studies, attempts were made to automate the sample processing steps for increased test capacity and speed up the analysis process using turbulent flow chromatography coupled to tandem mass spectrometry (TFC-MS/MS). The study also used a relatively much larger number of specimens to validate their approach against standard RT-qPCR. The sensitivity and specificity of the assay were 84 and 97%, respectively, with an estimated test capacity of 500 samples per day (Cardozo et al., 2020).

**TABLE 2:** COVID-19 testing by mass-spectrometry.

Specimen	No of patients	Reference method	Specimen preprocessing	Target	*MS approach	Results	References
NPS, OPS	985	RT-qPCR	Ethanol precipitation, lysis, trypsin digestion; automated magnetic bead-based preparation	Nucleoprotein	TFC-MS/MS	Sensitivity: 84% Specificity: 97%	Cardozo et al., 2020
NPS	362	RT-qPCR	No preprocessing required, 1:1 mixing with matrix solution	Untargeted	MALDI-TOF MS; machine learning – SVM	Accuracy: 93.9%) FP: 7%, FN: 5%	Nachtigall et al., 2020
Gargle solution	3	RT-qPCR	Acetone precipitation, tryptic digestion	Nucleoprotein	LC-MS	SARS-CoV-2 nucleoprotein identified in 2 of the three samples	Ihling et al., 2020
NPS	8	RT-qPCR	Heating, isopropanol precipitation, tryptic digestion	Nucleoprotein	LC-MS/MS	N protein detection in SARS-CoV-2 positive samples	Nikolaev et al., 2020
NPS	311	RT-qPCR	No preprocessing required, 1 μl specimen mixed with 1 μl matrix solution	Untargeted	MALDI-TOF MS; Multivariate analysis	Accuracy:67.66%, Sensitivity:61.76%, Specificity:71.72%	Rocca et al., 2020
NPS, OPS	103	RT-qPCR	Lysis, TCA precipitation	Spike, replicase	LC-MS/MS	Sensitivity: 90.5% Specificity: 100%	Singh et al., 2020
Serum	298	RT-qPCR	Dilution, 1:1 mixing with matrix solution	Untargeted	MALDI-TOF MS; machine learning – logistic regression	Accuracy: 99% Sensitivity: 98% Specificity: 100%	Yan et al., 2021
NPS	199	RT-qPCR	No preprocessing required, 1:1 mixing with matrix solution	Untargeted	MALDI-TOF MS; machine learning –	Accuracy: 98.3%, PPA:100% NPA: 96%	Tran et al., 2021
NPS	16	RT-qPCR	Heat inactivation, denaturation, tryptic digestion	Nucleoprotein, spike	LC-MS/MS	94% concordance	Schuster et al., 2021
NPS	237	RT-qPCR	Virus inactivation with guanidine thiocyanate, dilution, 1:1 mixing with matrix solution	Untargeted	MALDI-TOF MS; machine learning	Accuracy, sensitivity and specificity: >90%	Deulofeu et al., 2021
Plasma	815	RT-qPCR	Dilution, homogenization, centrifugation, formic acid ionization	Untargeted	HESI-MS; machine learning	Sensitivity > 83% Specificity > 96%	Delafiori et al., 2021

RT-qPCR, reverse transcriptase quantitative polymerase chain reaction; NPS, nasopharyngeal swab; OPS, oropharyngeal swab; TCA, trichloroacetic acid; TFC-MS/MS, turbulent flow chromatography coupled to tandem mass spectrometry; MALDI-TOF MS, matrix assisted laser desorption ionization-time of flight mass spectrometry; LC-MS, liquid chromatography mass spectrometry; LC-MS/MS, liquid chromatography-tandem mass spectrometry; FP, false positive; FN, false negative; PPA, positive percent agreement; NPA, negative percent agreement; SVM, support vector machine; HESI-MS, heated electrospray ionization – mass spectrometry. * Studies with nucleic acid amplification tests (NAAT) based on mass spectrometry were excluded.

Compared to the targeted approach, detection of SARS-CoV-2 in clinical samples with untargeted, MALDI-TOF MS approach appeared more suitable in terms of the ease of sample preparation, because little or no pre-preprocessing is necessary. Although the chemical identities of the biomarkers used for SARS-CoV-2 detection remains unidentified by the untargeted approach, the use of various machine learning strategies to identify spectral signatures specific to COVID-19 has been shown to be very promising. For example, a method to detect SARS-CoV-2 in NPS using MALDI-TOF MS combined with support vector machine approach was described with an achievable accuracy of 93.9%, suggesting that their approach can be used to reliably detect SARS-CoV-2 in nasal swab samples (Nachtigall et al., 2020). Another study combined MALDI-TOF MS with multivariate analysis for the detection of SARS-CoV-2 virus in NPS but achieved a relatively lower rate of accuracy using similar number of specimens (Rocca et al., 2020). Other studies evaluated relatively lower number of specimens but showed significant potential for MALDI-TOF MS to be applied to detect SARS-CoV-2 as an alternative tests to RT-qPCR assays. Interestingly, in one of the studies, serum specimens were tested instead of NPS specimens with highly encouraging, yet preliminary performance data. The approach was unique in that molecular changes in host in response to SARS-CoV-2 infection instead of the virus itself was targeted. The study also partially annotated feature peaks representing COVID-19 by analyzing sera using LC-MS/MS. However, the study only included symptomatic patients with different levels of severity (Yan et al., 2021). Additional, independent studies may confirm the role of biomarkers identified in this study in COVID-19 diagnosis.

## Metabolomics in COVID-19 Prognosis

Although most patients with COVID-19 are either asymptomatic or have mild disease, approximately 14% patients may have severe disease and 5% patients may need critical care. Acute respiratory distress syndrome (ARDS) is the most common complication in patients with severe COVID-19 disease, but the disease does not necessarily remain localized to the respiratory tract, and can involve many other organs of the body. After viral entry into nasal epithelial cells with the help of host receptor ACE2, replication and propagation, the virus makes its way through upper respiratory tract to alveolar pneumocytes releasing many different cytokines and inflammatory markers. This is known as “cytokine storm” which attracts neutrophils, CD4 helper T cells and CD8 cytotoxic T cells causing inflammation and lung injury and eventually ARDS. Further propagation of the virus in lymphocytes and vascular endothelial cells and viremia may lead to exaggerated systemic inflammatory response, shock, or multiorgan dysfunction. The majority of these patients may suffer from acute cardiac, kidney, and liver injury, cardiac arrhythmias, rhabdomyolysis, coagulopathy, and shock. Although elderly people and patients with co-morbidities such as cardiovascular disease, diabetes mellitus, immunosuppression, and obesity are more likely to have severe disease, healthy persons of any age may become critically ill with COVID-19. While laboratory findings such as lymphopenia, thrombocytopenia, and elevated liver enzymes, LDH, CRP, D-dimer, PT, troponin, CPK, and inflammatory cytokines such as IL-6 and TNF-α and abnormal radiological findings are associated with worse outcome with the disease, these are non-specific and are widely variable (Huang et al., 2020; UpToDate, 2020; Wiersinga et al., 2020; Parasher, 2021). Therefore, a tremendous amount of work has been done over the past year to identify biomarkers to predict the outcome of the disease so that appropriate treatment and care can be given to patients who are severely ill with COVID-19. As such, a number of biomarkers have been identified according to different organ systems which have recently been reviewed (Samprathi and Jayashree, 2020).

Apart from studies specifically focusing on hematologic, biochemical and immunological biomarkers, a number of metabolomics studies have also been conducted revealing dysregulation in several metabolic pathways and their roles in COVID-19 pathogenesis. Some of the studies, which have reported metabolomics-based candidate prognostic biomarkers are summarized in Table 3. Majority of these studies applied LC-MS/MS methods for detection and identification of metabolites in serum or plasma samples, but GC-MS and NMR were also applied in some studies. Both targeted and un-targeted metabolomics approach were used leading to the identification of specific metabolites or predictor biomolecules and their association with COVID-19 severity. In a few studies, multi-omics approach was applied for integrated analysis of the proteome, lipidome, and metabolome. This is particularly important in the context that the severity of COVID-19 disease is linked to host inflammatory process, which may be related to alterations in cellular metabolic processes. This is supported by earlier reports that metabolic pathways are involved in the regulation of innate and adaptive immunity against various viral infection (Wang et al., 2020; Saez-Cirion and Sereti, 2021). In a recent immunometabolic study, strong association was seen between proinflammatory cytokines and chemokines, such as IL-6, M-CSF, IL-1α, and IL-1β with metabolites involved in amino acid metabolism, NAD+ metabolism, purine and pyrimidine metabolism, TCA cycle, and primary bile acid metabolism in severe COVID-19 patients (Xiao et al., 2021).

**TABLE 3:** Metabolomics based potential prognostic markers for COVID-19.

Specimen	No. of patients	Metabolomics approach (N)	Patient groups	Severity marker/criteria	Metabolic pathway/s affected	Potential metabolomic markers	Association	Strength/weakness	References
Serum	65	GC-MS (46)	Mild vs. severe COVID-19 patients	Respiratory failure, respiratory rate > 30 bpm, O_2_ saturation < 92%, PaO_2_/FiO_2_ < 300 mmHg52.	Valine and threonine catabolism	α-hydroxyl acids	Correlation with O_2_ saturation/lung damage FC: 1.8–2.3; adjusted *p* < 0.05	Limited data	Paez-Franco et al., 2021
Plasma	104	GC-MS UHPLC/MS (77)	COVID-19 PCR positive vs. negative patients with flu-like symptoms	Mild – symptoms, no CT scan or hospitalization Moderate – dyspnea, pneumonia by CT scan, hospitalization, O_2_ supplementation Critical – ICU admission, respiratory distress, intubation and mechanical ventilation	Kynurenine pathway	Anthranilic acid	Correlation with poor prognosis and high IL-10/18 (*R* = 0.55/0.46; *p* = 0.0092/0.037)	Correlation with known immunosuppressive role Small sample size	Danlos et al., 2021
Plasma	30	LC-MS/MS, NMR (162)	COVID-19 positive and negative ICU patients and healthy controls	ICU admission, mortality	–	Kynurenine Creatinine/arginine ratio	Prediction of COVID-19 associated death (accuracy = 100%)	KP pathway involvement confirmed in multiple studies Small sample size	Fraser et al., 2020
Serum	49	UHPLC-MS	COVID-19 positive and negative patients	Severity inferred from IL-6 levels, CRP and BUN	Tryptophan metabolism/kynurenine pathway	Acylcarnitines, kynurenine, and methionine sulfoxide	Positive correlation with IL-6 [–log(*p*) > 2]	KP pathway involvement confirmed in multiple studies Small sample size	Thomas et al., 2020
						Free fatty acid and tryptophan levels	Negative correlation with CRP [–log(*p*) > 2]		
						Acylcarnitines	Positive correlation with BUN [–log(*p*) > 2]		
Serum	187	GC-MS (75)	Mild vs. severe disease	Dyspnea, respiratory rate ≥ 30/min; O_2_ saturation ≤ 93%, PaO_2_/FiO_2_ < 300 mmHg52, lung infiltrates > 50%	–	2-hydroxy-3- methylbutyric acid, 3-hydroxybutyric acid, cholesterol, succinic acid, L-ornithine, oleic acid and palmitelaidic acid	Progression from mild to severe COVID-19 (AUC 0.969)	Free fatty acid changes observed in multiple studies Small sample size	Shi et al., 2021
Plasma	49	LC-MS/MS (221)	Moderate, severe and critical	O_2_ saturation, analytical parameters and radiological findings	Ceramides, tryptophan metabolism, and NAD-consuming reactions	Kynurenine/tryptophan, 3-hydroxykynurenine/kynurenine, and 3-hydroxykynurenine/tryptophan	Increase with COVID-19 severity (*p* ≤ 0.001)	KP pathway involvement confirmed in multiple studies Small sample size	Marin-Corral et al., 2021
Serum	61	Targeted: UHPLC-MS/MS (258) Untargeted: UHPLC quadruple TOF high-resolution MS/MS system (155)	Mild, severe COVID-19 patients and healthy control	Respiratory distress, respiratory rate ≥ 30/min; O_2_ saturation ≤ 93%, PaO_2_/FiO_2_ < 300 mmHg5	Nicotinate and nicotinamide metabolism, tryptophan metabolism, and citrate cycle	–	Correlation with IL-6, IP-10, and M-CSF	KP pathway involvement confirmed in multiple studies Supporting data from longitudinal study Small sample size	Xiao et al., 2021
Plasma	341 (700 samples – longitudinal over 3 months)	Untargeted: LC-MS/machine learning (235 polar metabolite and 472 lipid metabolite)	COVID-19 positive patients with different levels of severity and COVID-19 negative patients	Symptoms, hospital and ICU admission, mechanical ventilation, death	–	25 predictor metabolites	Predict disease severity	Results confirmed by animal testing Longitudinal study Predictor metabolites remained uncharacterized	Sindelar et al., 2021
Plasma	161	Untargeted: UPLC-MS/MS GCxGC-MS (2075 lipids and 500 small molecules)	Critical and non-critical COVID-19 patients and healthy controls	Mild to severe – O_2_ supplementation, no mechanical or non-invasive ventilation Critical – respiratory failure, ICU admission, mechanical ventilation	Gluconeogenesis and the metabolism of porphyrins	Arachidonic acid and oleic acid	Correlated to severity of the disease (AUC > 0.98)	Free fatty acid changes observed in multiple studies Small sample size	Barberis et al., 2020
Plasma	85	NMR, LC-MS/MS; Multi-omics (348)	Mild to severe vs. critical	Mild – clinical signs of pneumonia but without O_2_ support Severe – with O_2_ support using non-invasive ventilation, tracheal tube, tracheotomy assist ventilation, or ECMO	Lipoprotein metabolism	HDL1, HDL4, LDL1, LDL4, LDL5, VLDL5, ApoA1, triglycerides, cholesterol	Disease severity	Preliminary data on potential biomarkers	Chen et al., 2020
Blood, urine	30	LC-MS Multi-omics: proteome, amino acids and lipidome (1254 proteins 664 lipids)	Severe vs. non-severe	Common – symptoms, pneumonia Severe – respiratory distress, respiratory rate > 30 bpm, O_2_ saturation < 92% at rest, PaO_2_/FiO_2_ < 300 mmHg52. Critical – respiratory failure, mechanical ventilation, shock, ICU admission	–	21 lipids and 4 proteins	AUC: 0.993 to classify severe patients	Limited data	Li et al., 2021
–	96	Bioinformatics analysis of published metabolomics data of COVID-19	Non-severe and severe COVID-19 patients, healthy controls and non-COVID-disease controls	Mild – symptoms without pneumonia Typical – fever or respiratory tract symptoms with pneumonia Severe – respiratory distress, respiratory rate > 30 bpm, O2 saturation < 92% at rest, PaO_2_/FiO_2_ < 300 mmHg52 Critical – respiratory failure, mechanical ventilation, shock, ICU admission, other organ failure	Nucleic acid and amino acid metabolism	Taurochenodeoxycholic acid 3-sulfate, glucuronate and *N,N,N-*trimethyl-alanylproline betaine TMAP	Top classifier of severe disease (ROC 0.805)	*In silico* data, requires experimental and clinical validation	Chen et al., 2021

*N, number of metabolites assessed; PaO_2_/FiO_2_, partial pressure of arterial oxygen to fraction of inspired oxygen; GC-MS, gas chromatography mass spectrometry; LC-MS, liquid chromatography mass spectrometry; LC-MS/MS, liquid chromatography-tandem mass spectrometry; NMR, nuclear magnetic resonance; UHPLC-MS, ultra-high performance liquid chromatography mass spectrometry; UHPLC-MS/MS, ultra-high performance liquid chromatography tandem mass spectrometry; UPLC-MS/MS, ultra-performance liquid chromatography tandem mass spectrometer; GCxGC-MS, comprehensive two-dimensional gas chromatography mass spectrometry; ECMO, extracorporeal membrane oxygenation; FC, fold change; AUC, area under curve; ROC, receiver operating characteristic.*

Accurate classification of COVID-19 disease severity is critical in predicting the prognosis of the disease based on metabolomic markers. The criteria used for severity classification in COVID-19 metabolomics studies were variable. Most studies used clinical symptoms such as dyspnea, respiratory distress and other signs of pneumonia, radiological features, the requirements for O_2_ supplementation and mechanical ventilation and hospital or ICU admission to classify the stages of the disease. However, laboratory parameters associated with COVID-19 such as CRP, IL-6, and BUN were also used in a few studies. Although several metabolic pathways were shown to be affected by severe COVID-19 disease such as amino acid metabolism, energy metabolism and lipid metabolism, most common effect observed was in amino acid metabolism. In particular, the role of Kynurenine pathway (KP) of tryptophan metabolism was consistently observed in several independent studies. KP is responsible for the catabolism of tryptophan in mammalian cells that is not used in protein synthesis and is known for its connection with the immune system and neurological disorders. Tryptophan metabolism can be diverted from serotonin/melatonin pathway via KP toward the biogenesis of nicotinamide adenine dinucleotide (NAD). The first and rate-limiting step of KP is made by two enzymes: tryptophan 2,3-dioxygenase (TDO) or indoleamine 2,3-dioxygenase (IDO), among which IDO is recognized to link KP with the immune system because it is activated by cytokines and appears to exert some anti-inflammatory effects (Davis and Liu, 2015; Savitz, 2020). This is consistent with the new finding on the association of KP with COVID-19 disease severity. The activity of IDO inferred from kynurenine/tryptophan ratio were inversely related with IL-6 levels in COVID-19 (Thomas et al., 2020). Altered levels of kynurenine and tryptophan were observed in association with COVID-19 severity in several studies that were independent of each other (Table 3). An increase in the levels of other metabolites of KP such as anthranilic acid and 3-hydroxykynurenine seen in COVID-19 patients in other studies also supports the role of KP metabolites as potential prognostic markers of the disease.

In a few studies, untargeted metabolomics approach was applied to the plasma samples of COVID-19 patients with different levels of severity, occasionally in combination with the targeted approach. In a study using ultra-performance liquid chromatography/tandem mass spectrometry (UPLC-MS/MS) and bi-dimensional gas chromatography/mass spectrometry (GCxGC-MS) for lipidome and metabolome analysis, respectively, ICU versus non-ICU COVID-19 patients were compared showing that free fatty acids such as oleic acid and arachidonic acid are present at much higher levels in ICU-COVID-19 patients (Barberis et al., 2020). In another interesting study, which is currently published as a pre-print, plasma samples from COVID-19 patients collected over six longitudinal time points were analyzed for metabolomic profiling in an unbiased manner. The study applied LC-MS for both polar and lipid metabolites along with various machine learning approaches revealing 25 robust predictor metabolites for disease severity. In support of their data, most of these metabolites were also identified in a Syrian hamster SARS-CoV-2 model (Sindelar et al., 2021). Multi-omics approach in one study revealed significant changes in lipoprotein metabolism including key enzymes associated with the pathways and the levels of lipoprotein subclasses and their compositional components (Chen et al., 2020). Another study assessed proteome, amino acids and lipidome identifying 21 lipids and 4 proteins as potential biomarkers for the prediction of disease severity (Li et al., 2021).

## Perspectives

From public health point of view, early detection of positive COVID-19 cases is of crucial importance to prevent the transmission of the virus and to effectively manage the pandemic, and analytically, RT-qPCR is the most sensitive and specific method of detection. However, RT-qPCR based methods are yet unavailable for mass-scale screening programs in many resource-limited countries because of cost, facilities requirement, and technological limitations. Standard RT-qPCR tests also require collection of nasopharyngeal specimens, which is invasive by nature and requires personal protective equipment (PPE) and training of individuals who collects the specimens. Despite the turnaround time of RT-qPCR tests is faster than many other microbiological methods, it is still slower for field level applications. To this end, metabolomics-based methods, in particular, analysis of VOCs in exhaled air from suspected COVID-19 patients hold significant promise for large-scale, filed level testing. The use of expired air, sweat, saliva or used mask all are highly convenient specimens for testing in POC setting. Depending on the detection technology, the results of tests may be instant or is available in minutes. However, test performance compared to the standard tests is yet uncertain given the wide range of variability in the sensitivity and specificity of these tests seen in different studies. Because several large-scale trials are currently underway in different countries, more reliable data on the sensitivity and specificity of the tests is expected to be published soon. It can be expected that field tests based on human breathomics may at least be able to filter the population to reduce burden on RT-qPCR tests.

Among the MS based approaches, MALDI-TOF MS has shown significant potential for laboratory testing of SARS-CoV-2. Because of its widespread use in the microbiology laboratories for identification of bacteria and yeast, most laboratories are already equipped with MALDI-TOF MS systems. Also, MALDI-TOF MS based methods are cheaper and faster than RT-qPCR if they can be applied directly on specimens without needing to process specimens. Preliminary data on the performance of MALDI-TOF MS in combination with specific machine learning algorithms is highly encouraging. However, MS based methods must be standardized and validated in independent laboratories using a larger number of specimens. Commercial development of MALDI-TOF MS based methods may help preventing variation in test quality in different laboratory setting. Biosafety aspects of the test process should also be carefully assessed in order to ensure that laboratory staffs are not affected by the test process.

Compared to the standard approach, one major limitation of metabolomics approach in COVID-19 diagnosis is that the approach (excluding targeted MS) depends on the host response against the virus instead of the virus itself. Because host response can be highly variable, unless extensively validated in diverse population, the application of metabolomics in COVID-19 diagnosis may result in false positive or negative results.

From patient management point of view, the wide spectrum of severity, the systemic nature of the disease, and the unpredictability in the outcome of the disease have made the management of COVID-19 patients particularly challenging. At present risk factors such as age and co-morbidities such as cardiovascular disease, obesity, diabetes, lung diseases, kidney diseases, cancer etc. and non-specific, inflammatory and organ specific markers are used to predict the prognosis of the disease. Undoubtably, specific metabolomics-based markers in relation to clinical outcome will benefit patients with severe disease. However, in clinical setting, real-life examples of such biomarkers for infectious diseases are scarce. Although a great deal of effort was put into COVID-19 biomarker research and a number of potential biomarkers were identified, no large-scale trials were performed to reliably determine their usefulness in clinical setting.

While certain metabolites such as those involved in tryptophan metabolism via KP have shown high reproducibility as potential markers of severity in independent studies, they have not been extensively validated in large patient populations to adjust data for age, sex, ethnicity and other demographic factors and underlying co-morbidities. While majority of these studies compared metabolite levels between COVID-19 positive and negative patients or between severe and non-severe patients, no data is yet available for the asymptomatic patients. Furthermore, there is wide variability in the severity criteria used in different studies and the validation parameters including test methods and reference methods were not standardized. It may be interesting to study individuals longitudinally before and after COVID-19 infection and assess metabolite changes. However, large sample sizes are necessary to account for variation in individual host responses. Although it is understandable that performing untargeted metabolomics or targeted metabolomics to measure hundreds of metabolites on large number of samples is not easy, it is probably time now to focus on the most promising candidate metabolomic markers.

At the same time, it is important to develop simple analytical procedures to analyze the target metabolites so that the test can be implemented in both resource-rich and resource-limited setting. Once the methodology is set with satisfactory performance, in terms of analytical sensitivity, specificity and reproducibility, large-scale, prospective validation projects must be conducted to determine the positive predictive value and negative predictive value and the clinical utility of implementing such tests over the standard approaches.

## Conclusion

Research on the application of metabolomics in the diagnosis and prognosis of infectious diseases, in particular, respiratory viral infection has been greatly accelerated by the urgency of COVID-19 pandemic. If successful, diagnostic applications based on VOCs in expired air, sweat, saliva or used masks from COVID-19 patients may bring a great solution to the current challenges in community screening or airport screening programs that the world is facing now. MALDI-TOF MS may be helpful in reducing cost, improving TAT and expanding test capacity of COVID-19 test facilities. Microbiology laboratories which do not have molecular test facilities but have access to MALDI-TOF instruments may take the benefit of this technology and offer COVID-19 tests. The massive surge in COVID-19 cases worldwide and the rates of severe disease and mortality associated with the disease have put the world in a highly challenging situation and triggered an unprecedented effort in biomarker research. The severity of COVID-19 is associated with perturbations in various metabolic pathways that are directly or indirectly linked to the systemic inflammatory response observed in severe COVID-19 patients. In particular, persistent findings on altered levels of KP metabolites such as tryptophan, kynurenine and 3-hydroxykynurenine points toward the roles of these metabolites in accurately predicting the course of COVID-19 disease. Therefore, method development and large-scale clinical trials involving these metabolites are necessary for successful implementation of novel, specific metabolic markers for the prognosis of COVID-19. Nevertheless, the progress that have been made in metabolomics research in relation to the diagnosis and prognosis of COVID-19 is enormous, compared to the similar research conducted on other infectious diseases during the past decade, and the lessons learned from COVID-19 may serve as a great resource for future applications of metabolomics in infectious disease diagnosis and management.

## Author Contributions

MH conceived the idea, reviewed the literature, and drafted the manuscript. MS and AP-L reviewed the literature, wrote part of the manuscript, and revised the manuscript. All the authors read and approved the final manuscript.

## Conflict of Interest

The authors declare that the research was conducted in the absence of any commercial or financial relationships that could be construed as a potential conflict of interest.

## Publisher’s Note

All claims expressed in this article are solely those of the authors and do not necessarily represent those of their affiliated organizations, or those of the publisher, the editors and the reviewers. Any product that may be evaluated in this article, or claim that may be made by its manufacturer, is not guaranteed or endorsed by the publisher.

## Footnotes

1 https://clinicaltrials.gov/
